# Organic Field‐Effect Transistors as Flexible, Tissue‐Equivalent Radiation Dosimeters in Medical Applications

**DOI:** 10.1002/advs.202001522

**Published:** 2020-07-30

**Authors:** Andrew M. Zeidell, Tong Ren, David S. Filston, Hamna F. Iqbal, Emma Holland, J. Daniel Bourland, John E. Anthony, Oana D. Jurchescu

**Affiliations:** ^1^ Department of Physics and Center for Functional Materials Wake Forest University Winston‐Salem NC 27109 USA; ^2^ Department of Radiation Oncology Wake Forest School of Medicine Wake Forest University Winston Salem NC 27157 USA; ^3^ University of Kentucky Center for Applied Energy Research Lexington KY 40511 USA

**Keywords:** dosimetry, flexible electronics, organic field effect transistors, radiation detection, X‐ray detection

## Abstract

Radiation therapy is one of the most prevalent procedures for cancer treatment, but the risks of malignancies induced by peripheral beam in healthy tissues surrounding the target is high. Therefore, being able to accurately measure the exposure dose is a critical aspect of patient care. Here a radiation detector based on an organic field‐effect transistor (RAD‐OFET) is introduced, an in vivo dosimeter that can be placed directly on a patient's skin to validate in real time the dose being delivered and ensure that for nearby regions an acceptable level of low dose is being received. This device reduces the errors faced by current technologies in approximating the dose profile in a patient's body, is sensitive for doses relevant to radiation treatment procedures, and robust when incorporated into conformal large‐area electronics. A model is proposed to describe the operation of RAD‐OFETs, based on the interplay between charge photogeneration and trapping.

## Graphical abstract

A radiation detector based on an organic field‐effect transistor (RAD‐OFET) is introduced for passive radiation dosimetry. RAD‐OFETs are tissue‐equivalent devices that can be placed directly on a patient's skin to directly measure radiation dose delivery and are sensitive for doses relevant to many radiation treatment procedures.

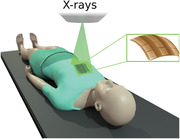

Cancer is one of the most common causes of death, with alarming growth rates worldwide, but fortunately can be treated with early diagnosis and care. Over the past decades, heroic research efforts focused on elucidating the causes and developing effective treatments for this disease, with chemotherapy, radiation therapy and surgery emerging as the leading approaches for treating cancer patients. In radiation therapy, one of the most common procedures, high‐energy ionizing photon and electron radiation is used to destroy or reduce the growth of cancer cells. Most modern treatment systems utilize linear accelerators to generate beams of electrons or X‐rays in the megavolt (MV) range, radioisotopes that produce gamma rays, or, more recently, cyclotrons to produce high energy protons.^[^ ^1^ ^]^ One of the biggest challenges in radiation oncology is to deliver the radiation only to the regions were the malignant cells are located, since the ionizing radiation can injure healthy tissues surrounding the target volumes.^[^ ^2^ ^]^ Many techniques have been implemented for maximizing the target dose, while minimizing normal tissue dose–this balance is always considered when planning a patient's radiation regimen. Radiation treatment regimens include high‐dose single fraction (e.g., stereotactic radiosurgery (SRS)), hypofractionated (stereotactic body radiation treatment (SBRT)), conventional fractionated (3D conformal radiation treatment (3D‐CRT), and intensity modulated radiation treatment (IMRT)) techniques that encompass considerations for physical and biological optimization criteria. Being able to measure the absorbed dose with high accuracy and sensitivity is therefore critical to successful patient treatment and long‐term survival. In contemporary clinical routines, first the beam is shaped, and the dose distribution is measured using a cylindrical diode array inside a poly(methyl methacrylate), (PMMA), phantom and then compared to the calculated dose distribution from a planning system, a quality assurance process that takes place a priori, during a treatment planning phase. Modern computers and machine learning techniques aid technicians in estimating the dose distribution within the patient by accounting for differences in geometry and composition between the patient and the detector, but unfortunately uncertainties persist.^[^ ^3^, ^4^ ^]^ In addition, positioning of the patient and even minor movements (e.g., breathing), can alter the received dose and targeted volume, and thus affect the outcome of the procedure. During the therapy session, electronic portal imaging devices (EPIDs) are used to ensure accurate beam placement, but the large number of variables involved in the post‐processing of the data can result in significant errors in the approximated dose profile in a patient's body due to inhomogeneities in the target (muscles/bones/adipose tissue), and person‐to person variations in the patient's anatomic structures. Alternative in vivo dosimeters were developed to validate the dose being delivered and ensure that for nearby regions an acceptable level of low dose is being received. Metal‐oxide semiconductor field‐effect transistors (MOSFETs) and photodiodes based on inorganic semiconductors (e.g., Silicon) can approximate the dose at the site of irradiation, however these devices can be invasive since they often rely on implantation into patient. The solution to these problems is placing the detector directly at the point where the radiation interacts with the human body, similar to a smart bandage (**Figure** **1**a). This approach can greatly enhance the accuracy of the measurements by eliminating the need for extensive data processing. At the same time, it will reduce the complexity and cost of the instruments and, therefore, the treatment. Here, we report on such radiation dosimeters based on organic field‐effect transistors (OFETs), called “RAD‐OFETs” (Radiation Detectors based on Organic Field‐Effect Transistors). These detectors take advantage of the key properties of organic semiconductors (OSCs): they are lightweight, conformal to the curvature of the human skin surface (Figure 1b), and thin enough to be placed at the site of irradiation without disturbing the delivered dose. The similarity in the density of organic compounds and human tissue, given by the comparable atomic numbers (*Z*) (Figure 1c), minimizes the correction factor for estimating the dose applied to the patient. We focus on energies ranging between 0 to 6 MeV and obtain sensitivities as high as (5.2 × 10^7^ ± 0.3 × 10^7^) µC Gy^−1^ cm^−3^ in RAD‐OFETs fabricated on flexible substrates, with an exceptional robustness upon bending. The RAD‐OFET dosimeters function by monitoring the shift in the threshold voltage of the OFETs and we propose a model that describes the physical mechanism responsible for the observed changes. The model is based on photogeneration of charges upon X‐ray exposure, followed by a distinct response to interaction with radiation of the electrons and holes, respectively. We find that the electron trapping at the semiconductor/dielectric interface occurs regardless of the absorbed dose. On the contrary, the dynamics of hole transport is given by the accumulation due to photoelectric effect and trapping by defects generated in the organic semiconductor film, a process which is dependent on the radiation dose. Our medical‐grade X‐ray dosimeters offer high sensitivity and ease of operation, while also taking advantage of the simple processing of organic materials, where large‐area arrays can be easily fabricated on flexible substrates using solution‐coating manufacturing techniques. These results provide a simple, yet effective and cost‐efficient solution for detection of ionizing radiation, which may yield new products for medical diagnostics and treatment dose measurement using low‐cost large‐area portable or wearable detectors. The versatile chemistry of organic compounds allows for fine tuning of the atomic number *Z* by modifications in the molecular structure to match the *Z* of any target material, whether it be bone, fat (adipose) tissue, or muscle tissue, therefore making this technique suitable for dose monitoring during imaging and treatment of a wide variety of medical radiation procedures.^[^ ^5^ ^]^ These results uncover new opportunities for organic circuits that will not only improve the quality of healthcare through better, lower cost in vivo dose monitoring during radiation therapy, but can also have applications into portable and wearable detectors or high energy radiation detectors for defense and space applications.

**Figure 1:**
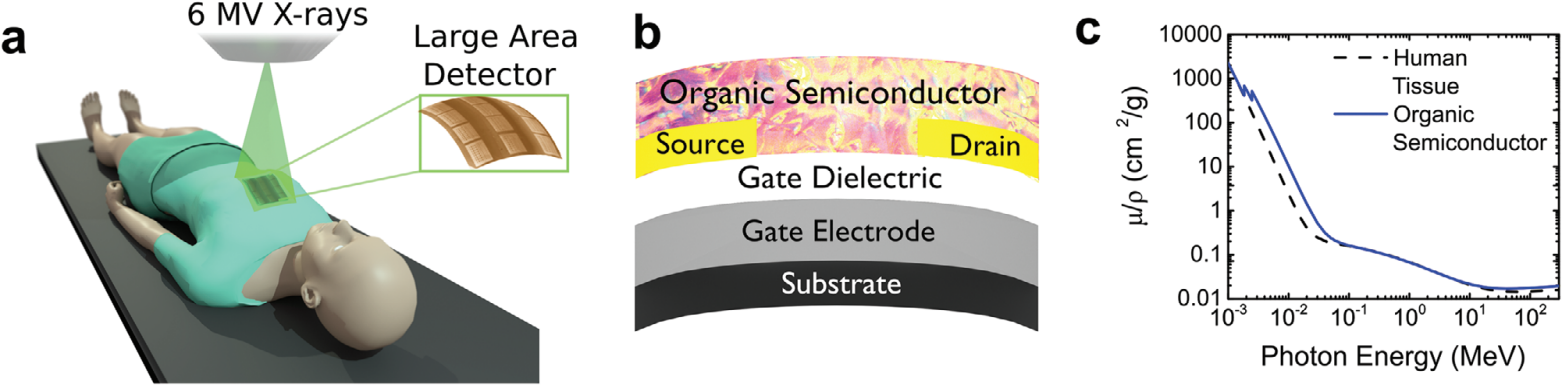
Flexible, large‐area radiation dosimeters based on organic transistors–RAD‐OFETs. a) Illustration of the placement of the RAD‐OFET on patients during radiation therapy, b) architecture of an individual RAD‐OFET sensing element, and c) comparison of the mass attenuation coefficient for human tissue and organic semiconductor over the large range of photon energies used for imaging and cancer treatment.

Organic semiconductors have drawn considerable attention in the medical field due to their inherent versatility in molecular design, their ease of processing, and biocompatibility. Devices made from OSCs can withstand temperatures used in sterilization,^[^ ^6^ ^]^ are stable in aqueous environments,^[^ ^7^ ^]^ and have been included in applications like heart rate monitors,^[^ ^8^ ^]^ and large area imaging arrays,^[^ ^9^, ^10^, ^11^ ^]^ placed on biocompatible substrates.^[^ ^12^, ^13^ ^]^ The use of biocompatible substrates has enabled ultra‐lightweight sensors for bio‐interfacing, from sensors thin enough to be attached directly to heart tissues to act as an electro‐cardiogram,^[^ ^14^, ^15^ ^]^ to neural probes capable of controlling neurotransmitter delivery.^[^ ^16^ ^]^ Novel device design has also opened up pathways to electrochemical devices, which can be used to sense metabolites produced in the body and act as bio‐powered sensors operating off electrochemical reactions with human skin to sense glucose and peptides.^[^ ^17^, ^18^ ^]^ Radiation detection using organic materials is a developing area, with applications ranging from hazardous material identification and homeland security, to imaging, personnel safety or space applications.^[^ ^5^, ^11^, ^19^, ^20^, ^21^, ^22^ ^]^ Much of this work, however, has focused on high radiation doses, or charged particle detection. Here we focus on radiation doses between 0.1 and 10 Gy (1 Gy = 1 J kg^−1^), which are typical values for medical imaging and therapy: for example doses of 0.0005 cGy account for dental and limb X‐ray imaging, 0.01 to 0.2 cGy are used for torso imaging, 0.4 to 2 cGy for body CT scans, 2.5 cGy for positron emission tomography (PET) scans, while radiation treatment of tumors requires doses between 100 to 200 cGy per fraction.^[^ ^4^ ^]^ To the best of our knowledge, this is the first example of using organic thin‐film devices as dosimeters for radiation procedures used for cancer diagnosis and treatment.

The organic field‐effect transistors used in this work had a bottom‐gate, bottom contact geometry.^[^ ^23^ ^]^ This device architecture allows for direct exposure of the organic semiconductor to the radiation beam. We started with a rigid structure, with a SiO_2_ layer playing the role of bottom‐gate dielectric, and later expanded to large‐area flexible substrates with a Cytop gate dielectric. The contacts were made of Au and the semiconductor was 2,8‐difluoro‐5,11‐bis(triethylsilylethynyl)anthradithiophene (diF‐TES ADT), a material that has shown remarkable electrical properties, with charge carrier mobilities close to 20 cm^2^ V^−1^ s^−1^ in devices with near ideal current–voltage characteristics,^[^ ^24^ ^]^ and strong sensitivity to X‐rays, although past work has focused on much higher doses and used different methods for detection.^[^ ^5^, ^25^ ^]^ The OFETs were fabricated using standard procedures and their electrical properties were first characterized in air and dark, prior to exposure.^[^ ^24^ ^]^ The devices were sandwiched without compression between two slabs of tissue‐equivalent calibration layers and irradiated through the top layer. The experimental set‐up is shown in **Figure** **2**a. The X‐ray radiation was supplied by a medical‐grade linear accelerator (Elekta Versa HD, Wake Forest University Baptist Medical Center), and the corresponding relative dose as a function of depth is shown in Figure 2b. Test samples consisting of arrays of OFET devices were placed in the center of the X‐ray field, as described in the Experimental Section. This setup reproduces the reference irradiation geometry used for radiation beam calibration in the clinical setting. The samples were exposed to radiation doses in increments of 0.2 Gy between 0 and 1 Gy, and in increments of 2 Gy from 2 to 10 Gy, at a nominal dose rate of 6 Gy min^−1^. Control samples were fabricated using identical procedures, but were not exposed to X‐rays, and were tested along with the irradiated ones, to be able to decouple the aging effects from the interaction with radiation (see Figure S1, Supporting Information).

**Figure 2:**
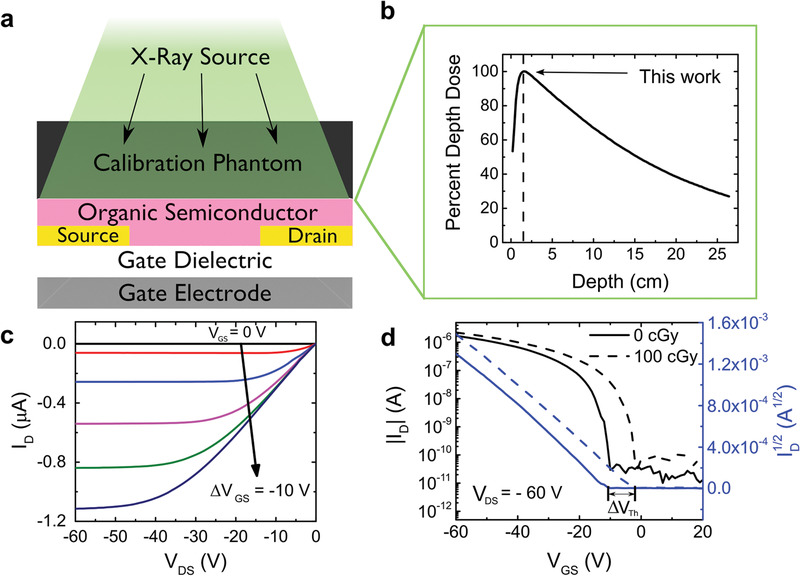
Experimental setup for radiation exposure and current‐voltage characteristics of a RAD‐OFET. a,b) Illustration of the experimental setup (a), the tissue equivalent phantom slab above the OFET ensures a calibrated dose (b) is delivered to devices during the experiment. c) Typical *I*–*V* characteristics of an OFET such as the ones used in this study. d) The threshold voltage shift induced by radiation exposure in a device. The geometry for this device was *L* = 90 µm and *W* = 800 µm.

Typical device output characteristics are shown in Figure 2c, where we plot the drain current *I*_D_ as a function of drain–source voltage *V*_DS_ for fixed gate–source voltages, *V*_GS_. In Figure 2d we show the dependence of *I*_D_ on *V*_GS_ at constant *V*_DS_ before exposure to radiation (solid line, see Figure S2 in the Supporting Information for forward and reverse sweeps) and after absorbing 100 cGy of radiation (dashed line); the curves obtained after other exposure doses are included in Figure S3 in the Supporting Information. It can be clearly observed that irradiation caused a shift in the threshold voltage of the OFET, i.e., the intercept of square‐root‐of‐drain‐current curve (blue) and the *V*_GS_‐axis, as marked in Figure 2d (more details about the analysis can be found in Figure S4, Supporting Information). This shift Δ*V*_Th_ is described by Equation (1)

(1) $$\Delta V_{\mathrm{Th}}=V_{\mathrm{ThD}}-V_{\mathrm{Th}0}$$

where *V*_ThD_ represents the threshold voltage at the target dose and *V*_Th0_ the threshold voltage at zero dose, i.e., before irradiation. To ensure that the threshold voltage was uniformly determined for each measurement, we adopted the method of the second derivative of *I*_D_ with respect to *V*_GS_.^[^ ^24^ ^]^ This type of dosimetry, where the measurements are taken after exposing the sample to radiation, is referred to as passive. Passive sensors rely on the detection of changes induced in the sample upon exposure to radiation and that persist after exposure; on the contrary, active sensors monitor in real time by recording the photoconductive gain.^[^ ^26^ ^]^ In this case, a passive detection ensures that the irradiation and device testing take place sequentially, i.e., there is no voltage applied to the RAD‐OFET while it is placed on a patient.

In **Figure** **3**a we plot the shift in the threshold voltage as a function of radiation dose, in red, along with the changes recorded in the control sample. While the control samples exhibit negligible changes, a shift in the threshold voltage can be observed upon irradiation, with two regions being clearly distinguished. At low irradiation doses, Region I—between 0 to 1 Gy, the irradiated OFETs exhibited a positive shift in the threshold voltage, up to a value of 6.1 ± 0.2 V, obtained at 1 Gy. The average sensitivity of the detector, defined as *S*_v_ = ∆*V*_Th_/Dose, was determined to be *S*_V_ = 60 ±^ ^8 mV cGy^−1^ for region I, a value which corresponds to *S*_I_ = (2.2 × 10^7^ ± 0.2 × 10^7^) µC Gy^−1^ cm^−3^, where *S*_I_ = (*I*_D_ − *I*_D,0_)/(Detector Volume × Dose Rate), *I*_D_ is the current measured after exposure, and *I*_D,0_ is the current prior to irradiation (both currents correspond to an applied gate–source voltage of *V*_GS_ = −60 V). This sensitivity value is similar to that obtained at high doses with other dosimeters based on organic semiconductors,^[^ ^5^, ^11^ ^]^ but in our case the performance was obtained under clinically relevant conditions for radiation treatment, where typical doses applied to patients do not exceed 200 cGy per treatment. In Region II, from 1 to 10 Gy, a negative shift in the threshold voltage is observed, with a sensitivity of *S*_V_ = 4.6 ±^ ^0.9 mV cGy^−1^ (*S*_I_ = (8.9 × 10^6^ ± 0.2 × 10^6^) µC Gy^−1^ cm^−3^).

**Figure 3:**
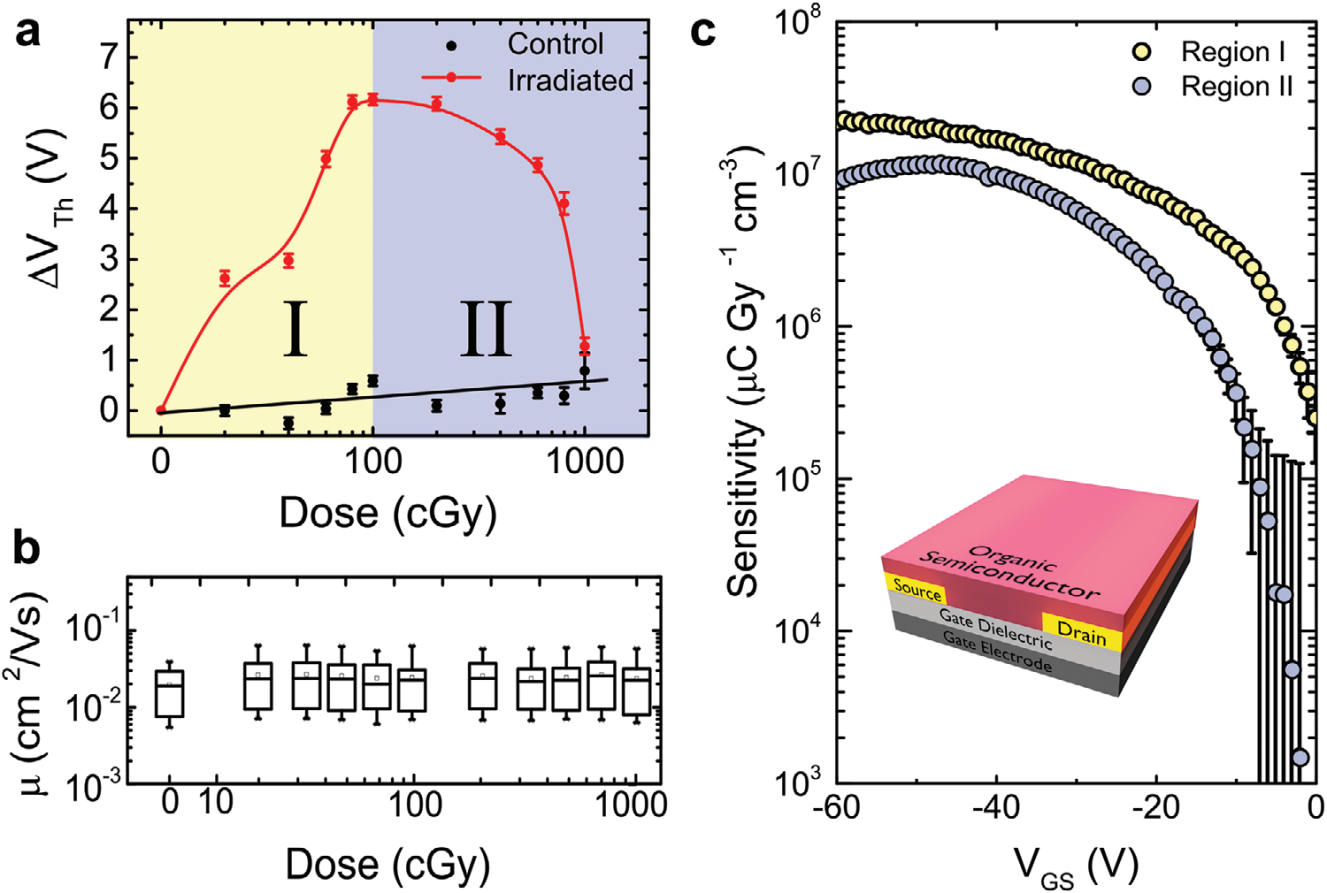
OFET response as a function of radiation dose. a) Threshold voltage shift versus applied radiation dose for the irradiated samples (red) and the control devices (black). In region I (yellow area) the shift is positive, while in region II (blue area) the shift is negative for the irradiated sample. No significant changes were recorded for the control sample. b) Box plot showing that the charge carrier mobility remains unchanged as a function of dose. c) Average device sensitivity as a function of gate‐source voltage in the two dose regimes. Averages for threshold voltage shift and mobility were performed over a sample size of *n* = 10.

To gain more insight into the processes responsible for the observed threshold voltage shifts, we also evaluated the evolution of the charge carrier mobility (*μ*) in response to sample irradiation. The mobility was calculated from the slope of the square‐root of the drain current curve with respect to the gate–source voltage (blue curves in Figure 2d), using Equation (2)

(2) $$I_{D}=\frac{W}{L}\frac{C_{i}}{2}\mu {\left(V_{\mathrm{GS}}-V_{\mathrm{Th}}\right)}^{2}$$

where *L* and *W* are the channel length and the channel width, respectively, and *C*_i_ the capacitance per unit area of the dielectric.^[^ ^23^ ^]^ Figure 3b shows the response of *μ* to cumulative dose; it is notable that the mobility did not change over the course of the treatments, suggesting that the organic semiconductor layer does not experience major chemical changes. Indeed, nuclear magnetic resonance spectroscopy, gas chromatography, UV–vis spectroscopy, and thin‐layer chromatography indicated that no degradation occurs in the organic semiconductor film. We do not exclude, however, that chemical changes take place, but the resulting impurity is below the detection limit of our techniques. Attempts to restore the device properties by thermal annealing or room‐temperature annealing resulted in partial recovery, while the solvent annealing results were inconclusive (see Figure S5, Supporting Information). The evolution of sensitivity with the gate‐source voltage for the low and high dose rates is included in Figure 3c in yellow and blue, respectively. At low bias the sensitivity is hampered by multiple trapping and de‐trapping events, which also impact the accuracy of reading, as demonstrated by the large error bars. When the voltage exceeds a critical value, which allows most of the holes accumulated in the transistor channel to be free, only a small dependence of the gate‐source voltage is recorded, thus the RAD‐OFET response is independent on the applied voltage.

The experimental results obtained upon controlled X‐ray irradiation of the OFETs allowed us to propose a mechanism for the operation of the RAD‐OFET dosimeters. The invariance in charge carrier mobility, along with partial recovery upon annealing, encouraged us to consider interfacial effects and structural defects as possible causes for the observed changes. The positive threshold voltage shift recorded upon exposure to low dose rates (Regime I) is indicative of electron trapping, a phenomenon that can occur either in the dielectric or in the organic semiconductor, close to the semiconductor/dielectric interface. The interaction of X‐rays with the OFETs results in a cascade of events. First, ionizing radiation causes the formation of an electron–hole pair, i.e., an exciton, in the organic semiconductor and in the SiO_2_ layers (see **Figure** **4**a).^[^ ^27^, ^28^, ^29^ ^]^ A fraction of these photogenerated charges will immediately recombine, while the rest will dissociate, resulting in free electrons and holes. The electrons are trapped at the semiconductor/dielectric interface by hydroxyl groups or other impurities and defects present here, while the holes, which are more resilient to trapping events, contribute directly to transport.^[^ ^30^ ^]^ There will be two contributions to the hole density: one from the processes occurring within the organic semiconductor layer, and the other from the dielectric, where the holes move through a series of hopping states toward the dielectric/semiconductor interface to contribute to transport (Figure 4a). The effect can be reduced if a high density of holes remains trapped in the oxide, a process that would contribute to a negative shift in the threshold voltage, which counteracts the effect of the holes that migrate to the interface and contribute to a positive shift. By performing capacitance measurements on the SiO_2_ dielectric layer (Figure 4b), we concluded that the hole trapping in the oxide is minimal, since the dielectric capacitance remained constant at the dose levels used in this study, at an average value of *C*_i_ = 17 ±^ ^1 nF cm^−2^. Some of the holes that reach the interface may, however, become deeply trapped here, creating long‐lived states which persist over longer time scales (e.g., days, months) and contribute to a negative shift in threshold voltage. While in Region I the photogeneration of holes overcomes the trapping events, resulting in a shift toward more positive threshold voltages, the higher doses (>100 cGy) experienced by the OFETs in Regime II appear to generate significant hole trapping and lead to a negative shift. This shift results from the eventual build‐up of deeply trapped holes at the interface and the structural defects occurring in the semiconductor, although we do not exclude the possibility of material degradation at levels below the detection limit of our chemical analysis techniques, but that are relevant to electronic transport.

**Figure 4:**
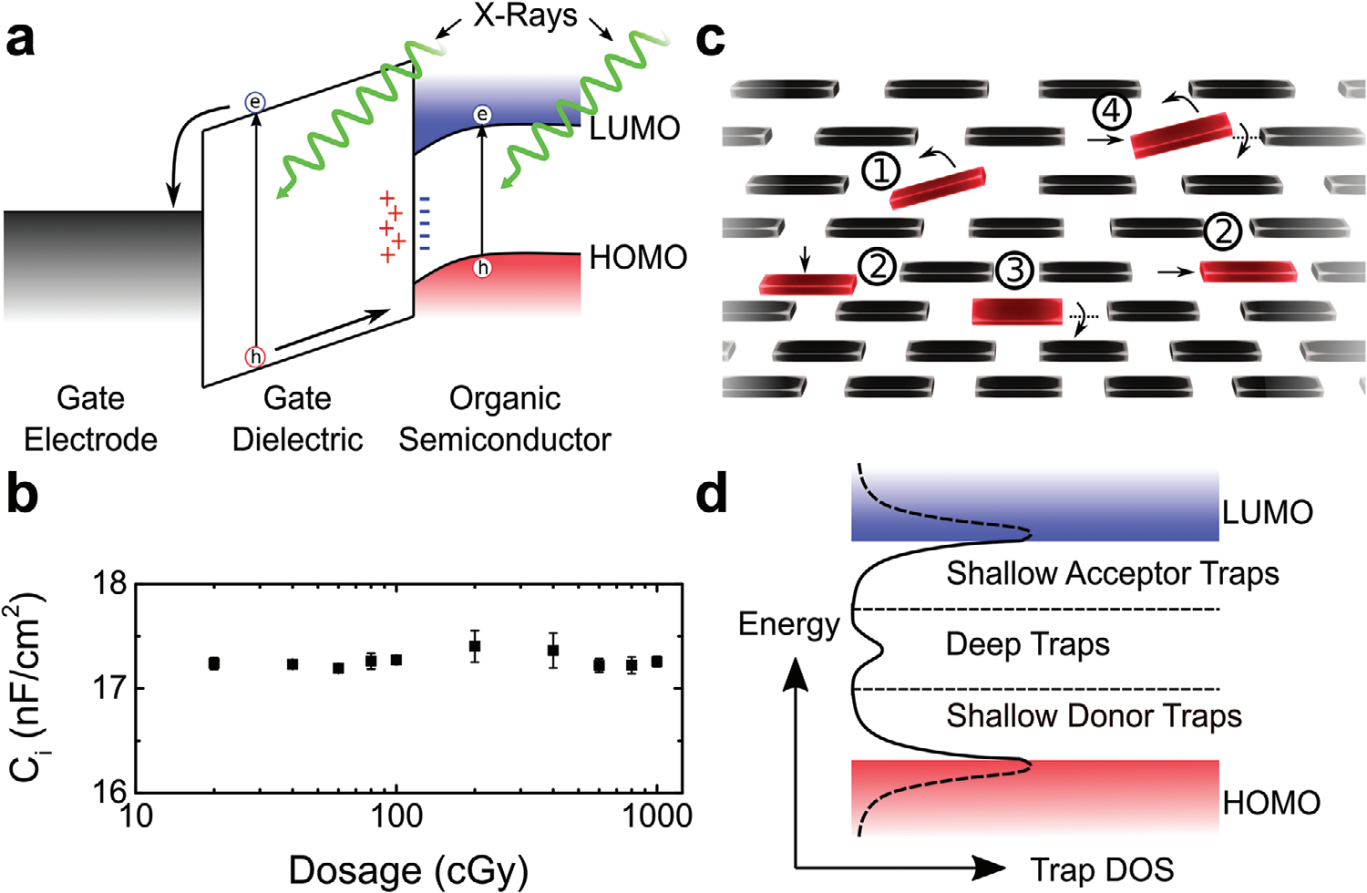
Mechanism for RAD‐OFET operation. a) Band energy diagram illustrating the generation of electron‐hole pairs when the device is irradiated, and the accumulation of deeply trapped holes at the interface. b) Capacitance measurements of the SiO_2_ dielectric suggesting that holes are not trapped in the bulk of the dielectric (sample size *n* = 10). c) Illustration of positional disorder in the diF‐TES ADT lattice, showing rotation (1,3), translation (2), and a mixture (4). d) The density of states in the bandgap of the organic semiconductor.

Trap generation in organic materials upon interaction with ionizing radiation has sparked great interest lately. Batlogg and co‐workers showed that proton irradiation results in cleaving of the C─H bond in single crystal rubrene, a process that generates deep trap states.^[^ ^31^ ^]^ They also found that X‐ray irradiation yields local structural disorder, which is a common source of traps in organic semiconductors.^[^ ^32^ ^]^ Proton irradiation of triisopropylsilyl ethynyl (TIPS) pentacene OFETs resulted in positional disorder,^[^ ^33^ ^]^ and electron irradiation of rubrene thin films induced n‐doping and a negative threshold voltage shift.^[^ ^34^ ^]^ Podzorov et al. used air‐gap rubrene OFETs to eliminate the interfacial affects and concluded that the shifts recorded in the threshold voltage upon interaction with X‐rays result from the deep traps created in the crystal.^[^ ^35^ ^]^ Figure 4c includes a sketch of possible molecular re‐orientations leading to positional disorder in the diF‐TES ADT film. The solid‐state packing was elucidated in our past work,^[^ ^36^ ^]^ and the distortion is exaggerated here for clarity: it includes rotations (1, 3), translations (2), and a combination of both (4). Such defects have been shown to be the origin of electronic traps with an energetic distribution that is dependent on the material and the exact nature of the defect.^[^ ^37^, ^38^ ^]^ A schematic representation of the energetic distribution of the trap density of states is included in Figure 4d, with shallow traps creating acceptor‐like and donor‐like tail states in the vicinity of the frontier orbitals and deep traps laying in the middle of the bandgap. Since we have not observed changes in charge carrier mobility upon exposure to radiation (Figure 3b), we hypothesize that the molecular re‐arrangements generate deep traps. De‐trapping of charge carriers residing here is highly unlikely since the trap depth is high, and thus they cannot contribute to transport.^[^ ^32^ ^]^

The tests performed on samples fabricated on silicon substrates provide robust perspectives for OFET adoption in medical radiation dosimetry. To take full advantage of the unique properties of organic semiconductors, next we integrated them with flexible substrates. Large‐area, flexible dosimeters can be placed directly onto the patient without the need for additional hardware, allowing for a higher accuracy and resolution of radiation detection and reducing the complexity and cost of the medical equipment. These devices were fabricated in a bottom gate, bottom contact configuration, onto polyethylene terephthalate (PET) flexible substrates with a predefined indium tin oxide (ITO) layer, which played the role of the gate electrode, and Cytop gate dielectric (**Figure** **5**a). Due to the hydrophobic nature of the fluorinated polymer Cytop, an additional surface treatment was applied to increase its surface energy and allow solution deposition of the organic semiconductor, as described elsewhere.^[^ ^39^ ^]^ These devices exhibited similar shifts in the threshold voltage, and the resulting sensitivity was (5.2 × 10^7^ ± 0.3 × 10^7^) µC Gy^−1^ cm^−3^. To test the mechanical robustness of the flexible OFET dosimeter, devices were flexed using a computer‐controlled servo (Figure 5c, inset) to a bending radius of 8 mm. Devices were exposed to a dose of 100 cGy and the sensitivity was measured as a function of the number of folding and unfolding cycles; the results for different effective gate‐source voltages are included in Figure 5, where the effective gate‐source voltage is the difference between the applied gate voltage and the device threshold voltage *V*_GS,*eff*_ = *V*_GS_ − *V*_Th_.

**Figure 5:**
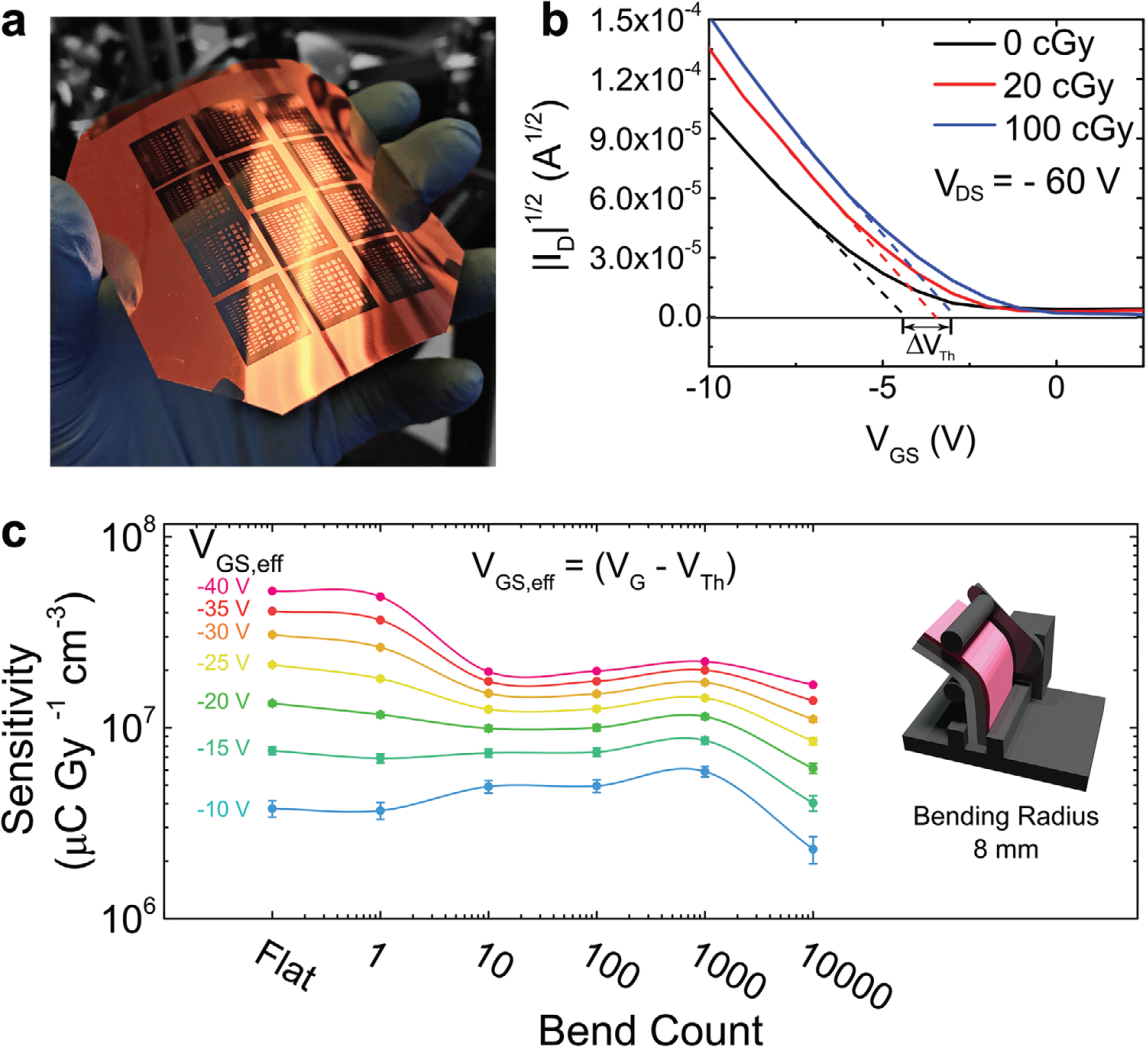
OFET radiation sensor on flexible substrates. a) Photo of an array of diF‐TES ADT OFET devices on PET/ITO substrates. b) Square root of *I*_D_ versus *V*_GS_ illustrating the shift in threshold voltages as a function of dose. The geometry for this device was *L* = 80 µm and *W* = 1000 µm. c) RAD‐OFET sensitivity after repeating bending cycles, for different effective *V*_GS_ (each point is an average of a sample size *n* = 12). The inset shows a schematic of the computer‐controlled bending set‐up.

The dependence of sensitivity on gate‐source voltage is in agreement with the results presented in Figure 3c. As for the tolerance to bending, a slight decrease in sensitivity occurs after 10 bends, most likely due to mechanical strains created at device interfaces and partial layer delamination, followed by a region of constant response between 10 and 10 000 bending cycles. A drastic decrease in sensitivity is observed after 10 000 cycles. Optical inspection of the films suggests that the micro‐cracks that formed in the film are responsible for the performance degradation. Nevertheless, the good tolerance to folding exhibited by our OFET radiation detectors for the first 1000 bending cycles makes them attractive for incorporation in large area flexible radiation detectors.

We introduced a new concept for fabricating passive radiation dosimeters for medical applications, which we named the RAD‐OFET. The device is based on organic thin‐film transistors, and the radiation dose is detected as a shift in the threshold voltage. The sensor is robust and highly sensitive for doses relevant to a variety of medical radiation procedures, including patient dose monitoring during cancer diagnosis and therapy. To understand the results, we proposed a model based on photogeneration of charges upon X‐ray exposure, followed by charge trapping at the semiconductor/dielectric interface. The hole dynamics is governed by a competition between the accumulation of the photogenerated holes and deep trapping within the organic semiconductor, a process which is highly depended on the radiation dose. For RAD‐OFETs fabricated on silicon substrates, at low doses, below 200 cGy, trapping is minimal, and a positive shift is recorded in the threshold voltage, with a sensitivity of 60 ±^ ^8 mV cGy^−1^ ((2.2 × 10^7^ ± 0.2 × 10^7^) µC Gy^−1^ cm^−3^). At high doses the trap formation prevails, leading to a negative shift and a sensitivity of 4.6 ±^ ^0.9 mV cGy^−1^ ((8.9 × 10^6^ ± 0.2 × 10^6^) µC Gy^−1^ cm^−3^). Solution processing allowed for integration with flexible substrates for the development of conformal RAD‐OFETs with high sensitivties, (5.2 × 10^7^ ± 0.3 × 10^7^) µC Gy^−1^ cm^−3^. Placement of the sensor directly onto the human body, coupled with the similarity in the atomic number between the electronically active layer and the human tissue, may greatly enhance the precision and reduce the complexity of the medical equipment, facilitating high quality measurement of patient doses. The application of therapeutic radiation with high precision increases the effectiveness on treating cancerous tissue and minimizes the impact on the surrounding healthy cells. The sensitivity of the proposed devices can be further enhanced with fine tuning the molecular structure of the organic semiconductor layer. These findings uncover new opportunities for organic circuits that will not only improve the quality of patient care, but they can have applications even beyond the cancer therapy. Such applications include OFET dosimeter placement in unique irradiation geometries and types, monitoring of personnel dose in hazardous environments, and measurement of space radiation.

## Experimental Section

##### Device Fabrication

Bottom‐gate, bottom‐contact devices were fabricated on both highly doped silicon substrates with a 200 nm SiO_2_ dielectric layer, and PET substrates with ITO gate electrodes and a protein‐modified dielectric layer, as described in earlier work.^[^ ^39^ ^]^ The SiO_2_ substrates were cleaned by immersion in hot acetone (85 °C) for 10 min, followed by a thorough rinse in acetone and immersion in hot isopropyl alcohol (IPA, 85 °C) for 10 min. The substrates were then rinsed in IPA and dried by a stream of nitrogen. Next, they were exposed to a UV–ozone treatment for 10 min, rinsed with deionized water, and dried in a nitrogen stream. For both substrate types, the source and drain electrodes were patterned by shadow masks and consisted of a 5 nm titanium adhesion layer deposited by electron beam deposition at a rate of 1 Å s^−1^, followed by thermally evaporated gold at a rate of 0.5 Å s^−1^. The contacts were treated for 30 min using a 30 × 10^−3^ m solution of room‐temperature pentafluorobenzene thiol (PFBT) in ethanol, rinsed with ethanol, and dried in a stream of nitrogen. The diF‐TES ADT semiconductor film was deposited by spin coating from a 16.5 mg mL^−1^ solution in chlorobenzene at 104 rad s^−1^ (1000 rpm) for 80 s, then placed in vacuum for 18 h to remove additional solvent. The CYTOP 809‐M dielectric layer was spin coated at 208 rad s^−1^ (2000 rpm) for 60 s, then annealed at 55 °C overnight, yielding a 1.4 µm film.

##### Device Characterization and Threshold Voltage Extraction

Transistor characterization was carried out in a nitrogen environment in dark using an Agilent 4155 C Semiconductor Parameter Analyzer. Devices were tested immediately after fabrication, as well as after each radiation dose. The control sample was not irradiated, but transported with and kept under similar environment as the irradiated samples. Capacitance measurements were taken using an Agilent E4980A LCR meter on reference and irradiated samples using the quasi‐static capacitance voltage measurement technique on samples consisting of the dielectric layer sandwiched between a gold top electrode and a highly doped Si substrate which also played the role of bottom electrode. Threshold voltages were extracted from the plot of the square root of the drain current, *I*_D_, with respect to the gate‐source voltage, *V*_GS_, using the maximum of the second derivative of *I*_D_ with respect to *V*_GS_ to bracket a linear extrapolation to where *I*_D_ = 0 V, and that voltage taken as the threshold voltage.

##### X‐Ray Irradiation of diF‐TES‐ADT Radiation Sensors

X‐ray radiation was administered to samples using an Elekta Versa HD linear accelerator at a dose rate of 600 cGy min^−1^. The devices were sandwiched without compression between two slabs of tissue equivalent material, i.e., (Gammex) Solid Water (top layer, to provide maximum percent depth dose to the devices, 1.5 cm thick; bottom layer, to provide backscatter to the devices, 10 cm thick) and irradiated through the top layer in increments of 20 cGy from 0 to 100 cGy, and then in increments of 200 cGy from 100 to 1000 cGy. Test samples, which were ≈1.5 cm × 1.5 cm and contained arrays of OFET devices, were placed in the center of a 10 × 10 cm^2^ field of 6 MV X‐rays, 100 cm from the source at depth of 1.5 cm.

##### Mechanical Durability Testing of Flexible Dosimeters

Repeated bending of devices was accomplished via a computer‐controlled servo to ensure a consistent curvature and the substrates were bent to a radius of 8 mm. Devices were electrically characterized for an irradiation dose of 100 cGy, before bending, and after each set of bending cycles, for sets of bending cycles from 1 to 10 000.

##### Chemical Analysis of Irradiated diF‐TES‐ADT

Analysis of irradiated samples by gas chromatography / mass spectrometry (Bruker 436 GC / Scion SQ MS) showed diF‐TES ADT as the dominant product, along with trace amounts (<0.1%) of the monofluoro and trifluoro derivatives, in the same ratio as in the original un‐irradiated sample. Proton NMR analysis of the irradiated sample (400 MHz Bruker Advance NEO) showed traces of endoperoxide, but this impurity often forms during sample preparation, and the same trace impurity was found when preparing pristine samples of diF TES ADT using the same conditions.

##### Statistical Analysis

The data used for the extraction of threshold voltage shifts was not pre‐processed before the analysis. The sensitivity of the dosimeters was extracted at constant effective gate‐source voltage, where the effective gate‐source voltage is the difference between the applied gate‐source voltage and the device threshold voltage *V*_GS,eff_ = *V*_GS_ − *V*_Th_. Device outliers were excluded prior to analysis based on the reliability of the *I*_D_^1/2 ^versus *V*_GS_ curve, using the coefficient of determination (*R* ^2^) value of a linear fit to the data. Values less than *R* ^2^ = 97% were excluded, as the model shown in Equation (2) predicts linear behavior of *I*_D_^1/2 ^versus *V*_GS_. Results are presented as (mean ± SD), where SD represents standard deviation, followed by the appropriate units. All experiments had a sample size of at least *n* = 10, and statistical analysis was performed using OriginPro 2016 (OriginLab Corporation).

## Conflict of Interest

The authors declare no conflict of interest.

## Supporting information

Supporting Information

## References

[1] J. Bernier , E. J. Hall , A. Giaccia , Nat. Rev. Cancer 2004, 4, 737. 15343280 10.1038/nrc1451

[2] W. D. Newhauser , M. Durante , Nat. Rev. Cancer 2011, 11, 438. 21593785 10.1038/nrc3069 PMC4101897

[3] D. Verellen , M. De Ridder , N. Linthout , K. Tournel , G. Soete , G. Storme , Nat. Rev. Cancer 2007, 7, 949. 18034185 10.1038/nrc2288

[4] L. Mullenders , M. Atkinson , H. Paretzke , L. Sabatier , S. Bouffler , Nat. Rev. Cancer 2009, 9, 596. 19629073 10.1038/nrc2677

[5] A. Ciavatti , L. Basiricò , I. Fratelli , S. Lai , P. Cosseddu , A. Bonfiglio , J. E. Anthony , B. Fraboni , Adv. Funct. Mater. 2019, 29, 1806119.

[6] K. Kuribara , H. Wang , N. Uchiyama , K. Fukuda , T. Yokota , U. Zschieschang , C. Jaye , D. Fischer , H. Klauk , T. Yamamoto , K. Takimiya , M. Ikeda , H. Kuwabara , T. Sekitani , Y. L. Loo , T. Someya , Nat. Commun. 2012, 3, 723. 22395614 10.1038/ncomms1721

[7] A. Giovannitti , C. B. Nielsen , D.‐T. Sbircea , S. Inal , M. Donahue , M. R. Niazi , D. A. Hanifi , A. Amassian , G. G. Malliaras , J. Rivnay , I. McCulloch , Nat. Commun. 2016, 7, 13066. 27713414 10.1038/ncomms13066 PMC5059848

[8] T. Yokota , P. Zalar , M. Kaltenbrunner , H. Jinno , N. Matsuhisa , H. Kitanosako , Y. Tachibana , W. Yukita , M. Koizumi , T. Someya , Sci. Adv. 2016, 2, 1501856. 10.1126/sciadv.1501856 PMC4846460 27152354

[9] P. C. Y. Chow , N. Matsuhisa , P. Zalar , M. Koizumi , T. Yokota , T. Someya , Nat. Commun. 2018, 9, 4546. 30382097 10.1038/s41467-018-06907-6 PMC6208338

[10] B. Fraboni , A. Ciavatti , F. Merlo , L. Pasquini , A. Cavallini , A. Quaranta , A. Bonfiglio , A. Fraleoni‐Morgera , Adv. Mater. 2012, 24, 2289. 22451192 10.1002/adma.201200283

[11] L. Basiricò , A. Ciavatti , T. Cramer , P. Cosseddu , A. Bonfiglio , B. Fraboni , Nat. Commun. 2016, 7, 13063. 27708274 10.1038/ncomms13063 PMC5059709

[12] D. T. Simon , E. O. Gabrielsson , K. Tybrandt , M. Berggren , Chem. Rev. 2016, 116, 13009. 27367172 10.1021/acs.chemrev.6b00146

[13] A. K. Bansal , S. Hou , O. Kulyk , E. M. Bowman , I. D. W. Samuel , Adv. Mater. 2015, 27, 7638. 25488890 10.1002/adma.201403560

[14] M. Kaltenbrunner , T. Sekitani , J. Reeder , T. Yokota , K. Kuribara , T. Tokuhara , M. Drack , R. Schwödiauer , I. Graz , S. Bauer‐Gogonea , S. Bauer , T. Someya , Nature 2013, 499, 458. 23887430 10.1038/nature12314

[15] S. Lee , Y. Inoue , D. Kim , A. Reuveny , K. Kuribara , T. Yokota , J. Reeder , M. Sekino , T. Sekitani , Y. Abe , T. Someya , Nat. Commun. 2014, 5, 5898. 25523614 10.1038/ncomms6898

[16] J. Rivnay , H. Wang , L. Fenno , K. Deisseroth , G. G. Malliaras , Sci. Adv. 2017, 3, 1601649. 10.1126/sciadv.1601649 PMC5466371 28630894

[17] J. Rivnay , S. Inal , A. Salleo , R. M. Owens , M. Berggren , G. G. Malliaras , Nat. Rev. Mater. 2018, 3, 17086.

[18] D. Ohayon , G. Nikiforidis , A. Savva , A. Giugni , S. Wustoni , T. Palanisamy , X. Chen , I. P. Maria , E. Di Fabrizio , P. M. F. J. Costa , I. McCulloch , S. Inal , Nat. Mater. 2020, 19, 456. 31844278 10.1038/s41563-019-0556-4

[19] H. M. Thirimanne , K. D. G. I. Jayawardena , A. J. Parnell , R. M. I. Bandara , A. Karalasingam , S. Pani , J. E. Huerdler , D. G. Lidzey , S. F. Tedde , A. Nisbet , C. A. Mills , S. R. P. Silva , Nat. Commun. 2018, 9, 2926. 30050037 10.1038/s41467-018-05301-6 PMC6062530

[20] S. Lai , P. Cosseddu , L. Basiricò , A. Ciavatti , B. Fraboni , A. Bonfiglio , Adv. Electron. Mater. 2017, 3, 1600409.

[21] M. A. Hupman , I. G. Hill , A. Syme , Radiat. Meas. 2018, 118, 31.

[22] S. Jain , S. G. Surya , P. K. Suggisetti , A. Gupta , V. Ramgopal Rao , IEEE Sens. J. 2019, 19, 4428.

[23] Z. A. Lamport , H. F. Haneef , S. Anand , M. Waldrip , O. D. Jurchescu , J. Appl. Phys. 2018, 124, 071101.

[24] Z. A. Lamport , K. J. Barth , H. Lee , E. Gann , S. Engmann , H. Chen , M. Guthold , I. McCulloch , J. E. Anthony , L. J. Richter , D. M. DeLongchamp , O. D. Jurchescu , Nat. Commun. 2018, 9, 5130. 30510263 10.1038/s41467-018-07388-3 PMC6277450

[25] L. Basiricò , A. Ciavatti , I. Fratelli , D. Dreossi , G. Tromba , S. Lai , P. Cosseddu , A. Bonfiglio , F. Mariotti , C. Dalla Val , V. Bellucci , J. E. Anthony , B. Fraboni , Front. Phys. 2020, 8, 1.

[26] H. N. Raval , V. R. Rao , IEEE Electron Device Lett. 2010, 31, 1482.

[27] T. M. Briere , A. S. Beddar , M. T. Gillin , Med. Phys. 2005, 32, 3346. 16370421 10.1118/1.2065447

[28] G. P. Beyer , G. G. Mann , J. A. Pursley , E. T. Espenhahn , C. Fraisse , D. J. Godfrey , M. Oldham , T. B. Carrea , N. Bolick , C. W. Scarantino , IEEE Sens. J. 2008, 8, 38.

[29] L. Carman , H. Paul Martinez , L. Voss , S. Hunter , P. Beck , N. Zaitseva , S. A. Payne , P. Irkhin , H. H. Choi , V. Podzorov , IEEE Trans. Nucl. Sci. 2017, 64, 781.

[30] L. L. Chua , J. Zaumseil , J. F. Chang , E. C. W. Ou , P. K. H. Ho , H. Sirringhaus , R. H. Friend , Nature 2005, 434, 194. 15758994 10.1038/nature03376

[31] T. Zimmerling , K. Mattenberger , M. Döbeli , M. J. Simon , B. Batlogg , Phys. Rev. B 2012, 85, 134101.

[32] H. F. Haneef , A. M. Zeidell , O. D. Jurchescu , J. Mater. Chem. C 2020, 8, 759.

[33] L. Basiricò , A. F. Basile , P. Cosseddu , S. Gerardin , T. Cramer , M. Bagatin , A. Ciavatti , A. Paccagnella , A. Bonfiglio , B. Fraboni , ACS Appl. Mater. Interfaces 2017, 9, 35150. 28925264 10.1021/acsami.7b08440

[34] J. J. Kim , J. M. Ha , H. M. Lee , H. S. Raza , J. W. Park , S. O. Cho , ACS Appl. Mater. Interfaces 2016, 8, 19192. 27399874 10.1021/acsami.6b05555

[35] V. Podzorov , E. Menard , A. Borissov , V. Kiryukhin , J. A. Rogers , M. E. Gershenson , Phys. Rev. Lett. 2004, 93, 086602. 15447211 10.1103/PhysRevLett.93.086602

[36] O. D. Jurchescu , D. A. Mourey , S. Subramanian , S. R. Parkin , B. M. Vogel , J. E. Anthony , T. N. Jackson , D. J. Gundlach , Phys. Rev. B 2009, 80, 085201.

[37] J. H. Kang , D. Da Silva Filho , J. L. Bredas , X. Y. Zhu , Appl. Phys. Lett. 2005, 86, 152115.

[38] P. J. Diemer , C. R. Lyle , Y. Mei , C. Sutton , M. M. Payne , J. E. Anthony , V. Coropceanu , J. L. Brédas , O. D. Jurchescu , Adv. Mater. 2013, 25, 6956. 24115382 10.1002/adma.201302838

[39] J. W. Ward , H. L. Smith , A. Zeidell , P. J. Diemer , S. R. Baker , H. Lee , M. M. Payne , J. E. Anthony , M. Guthold , O. D. Jurchescu , ACS Appl. Mater. Interfaces 2017, 9, 18120. 28485580 10.1021/acsami.7b03232

