# Supplementary material for: Organic Field‐Effect Transistors as Flexible, Tissue‐Equivalent Radiation Dosimeters in Medical Applications
Source: Adv Sci (Weinh). 2020 Jul 30;7(18):2001522. doi: 10.1002/advs.202001522 (PMC7509662; doi:10.1002/advs.202001522)
Supplement: Supplementary file 1 — Supporting Information [file ADVS-7-2001522-s001.pdf]

Copyright WILEY-VCH Verlag GmbH & Co. KGaA, 69469 Weinheim, Germany, 2020.

ADVANCED SCIENCE
Open Access

Supporting Information

for *Adv. Sci.*, DOI: 10.1002/advs.202001522

Organic Field-Effect Transistors as Flexible, Tissue-Equivalent Radiation Dosimeters in Medical Applications

*Andrew M. Zeidell, Tong Ren, David S. Filston, Hamna F. Iqbal, Emma Holland, J. Daniel Bourland, John E. Anthony, and Oana D. Jurchescu**

Copyright WILEY-VCH Verlag GmbH & Co. KGaA, 69469 Weinheim, Germany, 2018.

Supporting Information

Organic Field-Effect Transistors as Flexible, Tissue-Equivalent Radiation Dosimeters in Medical Applications

*Andrew M. Zeidell, Tong Ren, David S. Filston, Hamna F. Iqbal, Emma Holland, J. Daniel Bourland, John E. Anthony, Oana D. Jurchescu**

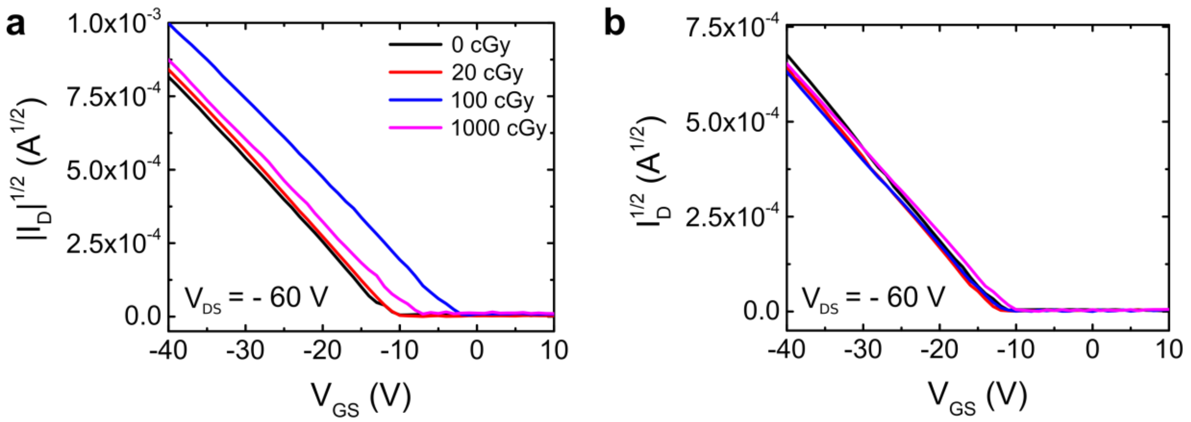

Figure S1. Comparison of threshold voltage shifts in irradiated sample and control sample. (a) Square root of the I_D vs V_{GS} for the irradiated and (b) the control sample.

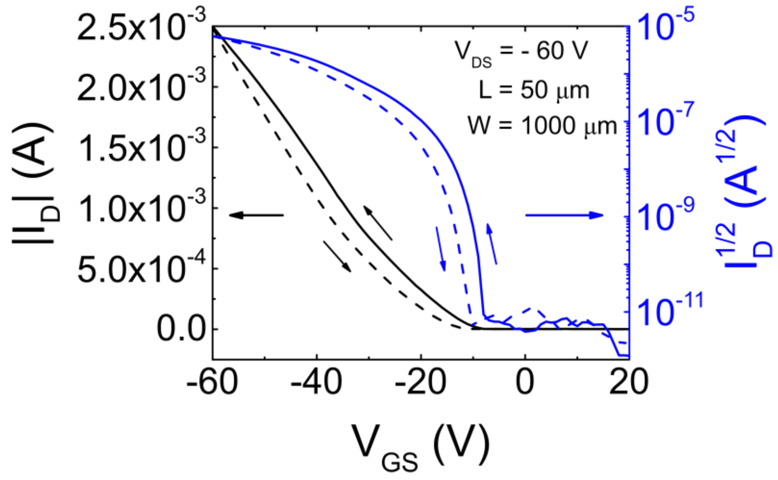

Figure S2. Forward (solid line) and reverse (dashed line) sweeps, showing I_D vs. V_{GS} curves (black) and $I_D^{1/2}$ vs. V_{GS} curves (blue) for a constant V_{DS} = - 60 V representative of the devices used in this study.

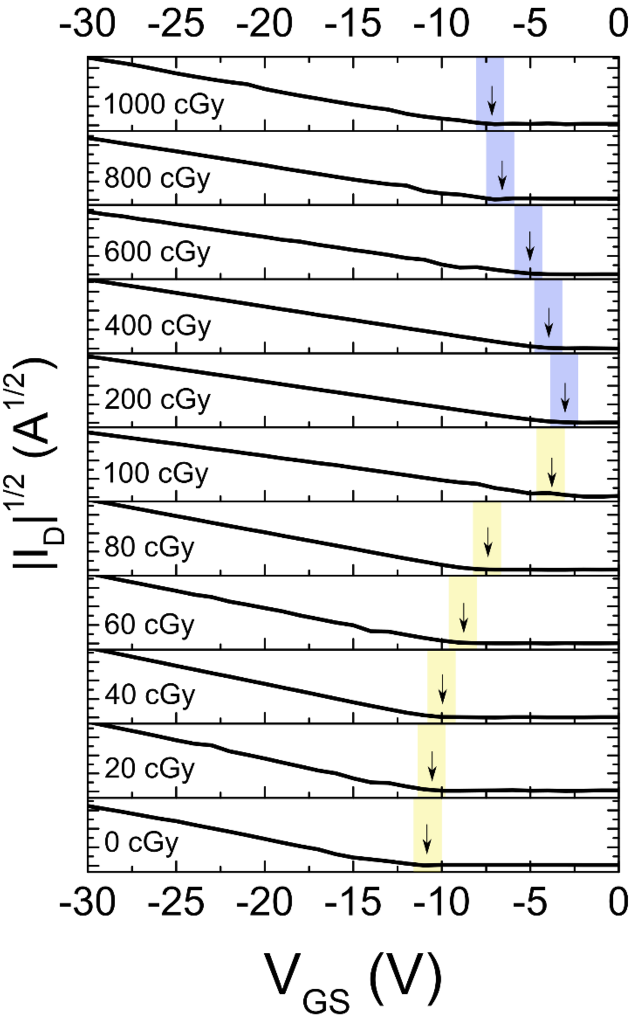

Figure S3. Representative $I_D^{1/2}$ vs. V_{GS} curves for a constant V_{DS} = - 60 V for all exposure doses. The threshold voltage, highlighted by the arrow, follows the trend observed in Figure 2. Note the largest shift was recorded at 200 cGy for this device, ΔV_{Th} of 6.5 V, followed by a decrease in ΔV_{Th} at higher doses.

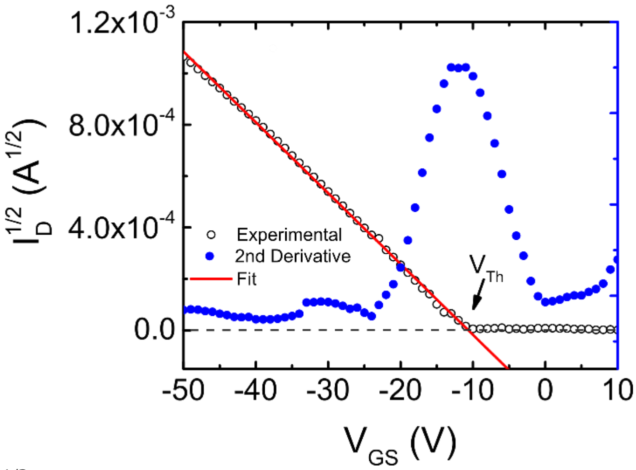

Figure S4. Example $I_D^{1/2}$ vs V_{GS} curve illustrating the method used to extract the threshold voltage. The maximum of the 2nd derivative of $I_D^{1/2}$ vs V_{GS} was used to fit a line to $I_D^{1/2}$, and the threshold voltage taken as the voltage where the fit line passes through the zero.

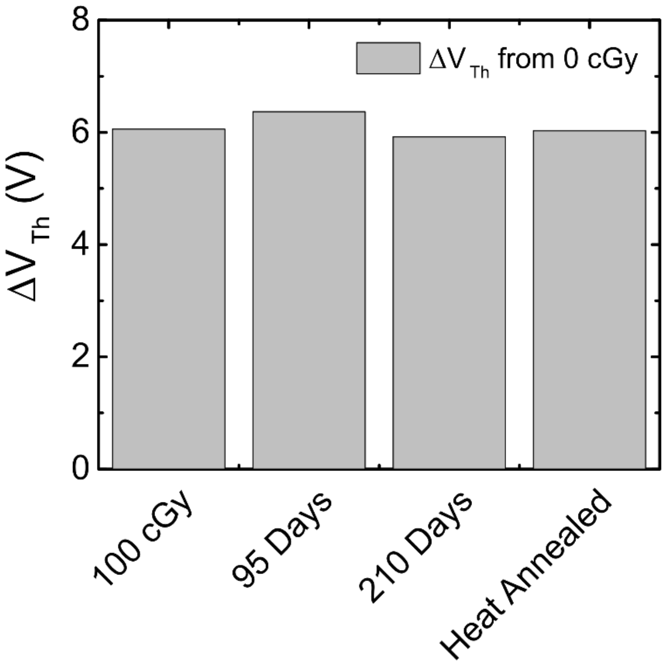

Figure S5. Partial recovery of the threshold voltage for various annealing times, and for high temperature annealing. Note that over time, at room temperature, the device recovered towards the 100 cGy value, while high temperature annealing (T = 75 °C) did not significantly affect the threshold voltage.
